# When hope falls short: differential roles of helplessness language and conditional expressions in predicting organizational future outlook

**DOI:** 10.3389/fpsyg.2026.1818679

**Published:** 2026-04-14

**Authors:** Joonghak Lee

**Affiliations:** Department of Business Administration, Dongguk University, Seoul, Republic of Korea

**Keywords:** BERT embedding, conditional expressions, hope theory, Korean organizations, learned helplessness, organizational future outlook, psychological contract, text mining

## Abstract

This study investigates why employees with equally low current job satisfaction develop markedly different expectations about their organization's future. Moving beyond static satisfaction perspectives, we examine the paradox of “same present, different futures.” Integrating psychological contract theory, hope theory, and learned helplessness theory, we conceptualize organizational future outlook as a temporally grounded indicator of the psychological contract and analyze how linguistic expressions reflect divergent cognitive orientations toward change. Using approximately 465,000 employee reviews collected from a Korean corporate review platform, we identified 93,276 reviews with uniformly low satisfaction ratings and applied a hybrid text mining approach combining dictionary-based analysis and BERT embeddings. Even within this uniformly dissatisfied group, future expectations diverged substantially: some anticipated growth, others stability, and many decline. Results indicate that the use of helplessness language (reflecting perceptions of stability, uncontrollability, and resignation) is systematically associated with more pessimistic future outlooks. In contrast, conditional expressions that articulate potential pathways for change (e.g., “if X changes, improvement is possible”) are associated with less pessimistic evaluations. Notably, the mere use of positive or hopeful vocabulary does not predict future outlook, suggesting that hope functions not as simple positive affect but as a cognitive capacity to imagine actionable pathways. These findings extend psychological contract theory by illuminating its temporal dimension and demonstrate how computational linguistic analysis can uncover subtle psychological mechanisms shaping employees' future-oriented organizational judgments.

## 1 Introduction

In recent years, South Korean organizations have faced unprecedented challenges. According to the Korea Labor Institute, employee satisfaction has steadily declined, with surveys indicating that over 60% of Korean employees report dissatisfaction with their current workplace conditions (Lee and Kim, 2023). High-profile corporate restructurings, stagnant wage growth, and intensifying work demands have contributed to a climate of organizational uncertainty. Despite these shared adversities, employees within the same organizations often diverge sharply in their expectations about the future, with some retaining hope for improvement while others resign themselves to perpetual stagnation. Understanding this divergence is not merely an academic exercise; it is a pressing practical concern for Korean organizations striving to retain talent and sustain commitment amid turbulence (Lee et al., 2019).

“Things are hard now, but next year will be better.”

This sentiment resonates with many employees navigating organizational difficulties. Yet this belief does not necessarily correspond to one's current level of satisfaction. Some employees who are deeply dissatisfied with their current job still maintain the expectation that “things will improve next year,” while others, equally dissatisfied, have already mentally withdrawn, resigned to the belief that “nothing will change anyway.” Same present circumstances, yet entirely different futures. Where does this paradox originate?

How organizational members perceive their current work experience, and, more importantly, how they project their organization's future, is a central concern in organizational behavior research. Traditionally, employee satisfaction research has focused on perceptions at a given point in time. However, a growing body of evidence suggests that employees' evaluations of their organization's future potential exert greater influence on turnover intentions, organizational citizenship behavior, and the maintenance of psychological contracts than current satisfaction levels alone (Ng and Feldman, 2012; Bal et al., 2013).

Of particular interest is the finding that even among employees reporting similarly low current satisfaction, future outlooks diverge dramatically. Some employees expect improvement (“things will get better next year”), while others express resignation (“nothing is going to change”). This divergence cannot be attributed solely to individual personality traits. Rather, how employees interpret their organizational environment and attribute the causes of existing problems may produce entirely different future outlooks even under objectively identical circumstances.

This study explores the psychological mechanisms through which currently dissatisfied employees form either optimistic or pessimistic views of their organization's future. Specifically, drawing on Snyder's (2002) hope theory and Seligman's (1975) learned helplessness concept, we analyze how “controllability language” and “resignation language” evident in employees' text messages to management are associated with future outlook evaluations.

The dataset comprises approximately 465,000 employee reviews collected from a leading Korean corporate review platform. These reviews contain unstructured text in which current and former employees freely describe their organizational experiences, enabling analysis of honest perceptions and emotions that survey-based research cannot easily capture. Crucially, we employ a hybrid text mining methodology combining BERT-based embeddings and dictionary-based analysis to quantitatively measure linguistic patterns of hope and helplessness from large-scale text data.

The central methodological contribution lies in the “same present, different futures” design. Whereas, prior research has examined correlations between current satisfaction and future outlook, this study holds current satisfaction constant and explores factors explaining variance in future outlook within this constrained sample. Specifically, we restrict the analytic sample to approximately 93,000 employees who rated their overall current satisfaction at 2 out of 5, and examine why some among this group expect “growth next year” while others expect “decline next year.”

Practically, our findings offer implications for how organizations should manage members' “beliefs about the future.” What is more dangerous than current difficulties is the extinction of future expectations. The moment employees believe “nothing will change anyway,” the psychological contract has already been exhausted, and physical departure is only a matter of time.

In sum, this study addresses the following research question: Among employees who share equivalently low current satisfaction, what linguistic and psychological mechanisms explain the divergence in their organizational future outlooks? Specifically, we examine (a) whether helplessness language (reflecting stable, uncontrollable attributions) is associated with more pessimistic future outlooks, and (b) whether conditional hope expressions (reflecting pathway thinking) buffer against such pessimism. By situating this inquiry within the Korean organizational context, where cultural norms of endurance and deferred gratification shape employees' temporal orientations toward their organizations, this study offers both theoretical and practical contributions to understanding how dissatisfied employees construct their future expectations.

## 2 Theoretical background and hypotheses

### 2.1 Organizational future outlook and psychological contract

The psychological contract refers to an individual's beliefs about the implicit mutual expectations and obligations between the employee and the organization (Rousseau, 1995). This concept extends beyond formal employment contracts to encompass employees' expectations of the organization and their trust that these expectations will be fulfilled. A defining characteristic of the psychological contract is its inherently future-oriented nature: employees commit to their organization with the expectation that current effort and dedication will be rewarded in the future, and they experience psychological contract breach when they perceive these expectations will not be met (Morrison and Robinson, 1997).

(Robinson and Morrison 2000) demonstrated that psychological contract breach is significantly associated with reduced organizational trust, decreased job satisfaction, and increased turnover intentions. However, even under objectively identical circumstances, there is substantial individual variation in the degree to which employees perceive contract violations. (Bal et al. 2008) argued that this variation depends on how employees evaluate the likelihood of future organizational change. Employees who believe the organization will improve despite current difficulties are more likely to maintain their psychological contract, whereas those pessimistic about the possibility of change are more likely to perceive contract violation.

In the Korean organizational context, the importance of future outlooks is particularly pronounced. Korean organizations have traditionally been characterized by cultural norms of endurance and deferred gratification, where employees tend to offset current dissatisfaction with expectations of future rewards (Hofstede, 2001; House et al., 2004). However, when this endurance fails to translate into actual improvement, accumulated dissatisfaction can culminate in abrupt departure. Understanding how employees project their organization's future thus carries special significance for Korean organizational management.

It is important to distinguish organizational future outlook from related but conceptually distinct constructs. First, unlike expected organizational performance, which focuses on objective forecasts of financial or strategic outcomes, organizational future outlook captures employees' subjective evaluations embedded within the psychological contract framework. Second, career optimism (Rottinghaus et al., 2005) pertains to individuals' expectations about their personal career trajectories, whereas organizational future outlook concerns the perceived trajectory of the organization itself. Third, future time perspective (Carstensen, 2006) reflects a general psychological orientation toward the future, while organizational future outlook is specifically anchored in employees' temporal appraisals of their organization's prospects. By clarifying these conceptual boundaries, we position organizational future outlook as a temporally grounded extension of the psychological contract that is distinct from general dispositional optimism or performance forecasting.

Furthermore, understanding Korean employees' future outlooks requires consideration of the broader sociocultural and ideological context shaping their organizational experiences. Korean society has historically been influenced by Confucian values emphasizing hierarchical relationships, loyalty, and collective harmony (Cho and Yoon, 2001). These values foster a tendency to justify current organizational conditions through a lens of long-term reciprocity, that is, the expectation that enduring present hardship will eventually be rewarded by the organization. Additionally, the concept of “nunchi” (social perceptiveness) and “jeong” (emotional bonding) in Korean workplaces may influence how employees frame their expectations about organizational futures (Kim and Park, 2003). Employees embedded in strong jeong-based relationships with their organizations may be more inclined to maintain hopeful future outlooks despite dissatisfaction, while those who perceive a breakdown in these cultural bonds may more readily adopt helplessness attributions.

### 2.2 Hope theory and its application in organizational contexts

Hope theory, as proposed by (Snyder 2002), conceptualizes hope not as a simple emotional state but as a cognitive component of goal-directed thinking. According to this theory, hope consists of two core elements. The first is “pathway thinking”: the cognitive appraisal that one can generate methods or routes for reaching a desired goal. The second is “agency thinking”: the belief that one has the motivation and ability to execute such pathways. Individuals high in hope can envision multiple routes toward goal attainment and, when faced with obstacles, can find alternative approaches and persist.

Applied to organizational contexts, employees high in hope would be expected to imagine specific ways in which the organization could improve and to believe that such improvements can be realized. (Luthans et al. 2007) included hope as a core component of Psychological Capital (PsyCap) and demonstrated that higher hope levels are positively associated with job performance, job satisfaction, and organizational commitment. (Peterson and Byron 2008) similarly found that employees high in hope generate more diverse solutions in problem-solving situations and recover more quickly from adversity.

A particular focus of this study is the linguistic expression of hope. Employees high in hope, when discussing the possibility of organizational improvement, are expected to employ conditional possibility language. Expressions such as “if things change, it will get better,” “if management is willing, change is possible,” or “if processes are improved, it can be done” indicate that the speaker is imagining pathways to change and believes such change to be achievable.

### 2.3 Learned helplessness and organizational resignation

Learned helplessness, as proposed by (Seligman 1975), explains the effects on motivation, cognition, and emotion of repeated experiences of uncontrollable negative events. The core mechanism is as follows: when an individual repeatedly experiences a lack of contingency between their actions and outcomes, they form the expectation that “nothing I do will make any difference,” which leads to a pervasive helplessness that extends to abandoning attempts even in future situations.

(Abramson et al. 1978) extended this framework by combining it with attribution theory to propose a reformulated model of learned helplessness. According to this reformulation, helplessness is exacerbated when negative outcomes are attributed to internal (self-blaming), stable (always this way), and global (pervasive across situations) causes. Applied to organizational contexts, expressions such as “this organization has always been like this,” “nothing will change anyway,” and “there's no point in saying anything” indicate that the speaker is attributing organizational problems to stable and uncontrollable causes.

(Martinko and Gardner 1982) were among the first to apply learned helplessness to organizational settings, arguing that repeated experiences of failure or uncontrollable organizational environments can generate demotivation and passive attitudes in organizational members. Subsequent research has documented that workplace helplessness is significantly associated with job burnout, depression, and turnover intentions (Ashforth, 1989).

In Korean organizational contexts, learned helplessness warrants particular attention. In environments dominated by hierarchical culture and authoritarian leadership, employees whose opinions are repeatedly ignored may develop organizational helplessness. Attitudes such as “there's nothing we can do about decisions made from above” or “just do as you're told” may represent expressions of learned helplessness commonly observed in Korean organizations (Hofstede, 2001; House et al., 2004).

### 2.4 The role of conditional hope expressions

A concept introduced in this study is “conditional hope expression.” This refers to linguistic expression patterns that discuss the possibility of future improvement contingent on the fulfillment of specific conditions, such as “if X changes, things will get better” or “if only Y is different, it can be done.” Such expressions can be interpreted as the linguistic manifestation of “pathway thinking” in Snyder's (2002) hope theory. They indicate that the speaker is imagining a specific pathway (a conditional change) toward the goal (organizational improvement) in the current situation.

The use of conditional expressions is distinct from simple positive language. Unlike vague optimism (“things will get better”), conditional expressions such as “if management communicates better, things will improve” indicate awareness of specific levers of change. This reflects that the employee perceives the organizational environment as controllable and believes that improvement is possible given specific changes.

In Korean, conditional expressions are grammatically and morphologically distinct, marked by specific verbal endings such as “~다면 (if/when),” “~라면 (if [hypothetical]), and “~한다면 (if [counterfactual]).” These endings linguistically mark that the speaker is positing a hypothetical situation. This study leverages these morphological features to automatically detect conditional expressions and analyze their relationship with future outlook.

(Tausczik and Pennebaker 2010) found that the use of conditional language is associated with causal reasoning ability and future-oriented thinking. Constructing a conditional statement requires the cognitive work of connecting the current state, a possible change, and its consequences. Thus, the use of conditional expressions goes beyond mere emotional expression to reflect analytical understanding of the situation and the capacity to construct future scenarios.

An important theoretical clarification concerns the relationship between conditional expressions and the two components of hope theory. While conditional expressions such as “if X changes, things will improve” most directly capture pathway thinking, that is, the cognitive appraisal of routes to desired goals, they may also implicitly reflect elements of agency thinking. By articulating a condition under which change is possible, employees may be signaling their belief that change agents (whether management, employees collectively, or the organization as a system) have the capacity to enact such conditions. However, we acknowledge that the current operationalization primarily captures pathway cognition rather than agency cognition. The conditional expression flag detects the identification of “routes for change” but does not directly measure the motivational belief that one can initiate or sustain effort along those routes. Future research could develop linguistic markers that distinguish between these two components [for example, distinguishing “if management changes policy, things will improve” (pathway) from “we can push for policy change” (agency)] to provide a more nuanced test of hope theory in organizational contexts.

Moreover, interpreting linguistic patterns in organizational texts requires attention to the social context in which language is produced. As (Boyd and Schwartz 2021) argued, natural language analysis must account for the social and situational factors that shape verbal behavior. In the Korean organizational context, the use of conditional expressions may be influenced by norms of indirect communication and face-saving, where employees express criticism or hope through hedged, conditional language rather than direct statements. This cultural dimension of language use underscores the importance of contextualizing linguistic markers within the specific sociocultural setting of the study, rather than assuming universal correspondence between linguistic forms and psychological states.

**Figure 1:**
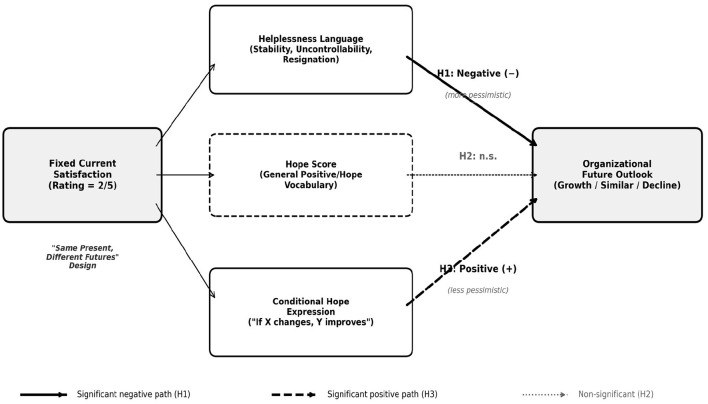
Conceptual framework: dual mechanisms of helplessness and conditional hope in predicting organizational future outlook.

Figure 1 presents the conceptual framework of this study, illustrating the dual mechanisms through which dissatisfied employees form divergent future outlooks. The framework depicts how, at a fixed level of current dissatisfaction, helplessness language (reflecting stable, uncontrollable, and global attributions) is associated with more pessimistic future outlooks, while conditional hope expressions (reflecting pathway thinking) are associated with less pessimistic outlooks. The framework also notes that general hope vocabulary, lacking the specificity of pathway thinking, does not predict future outlook, highlighting the importance of the cognitive structure of hope over its mere lexical presence.

### 2.5 Hypotheses

**Hypothesis 1:** At the same level of current job satisfaction, higher use of learned helplessness language will be associated with more pessimistic evaluations of organizational future outlook.

**Hypothesis 2:** At the same level of current job satisfaction, employees who use conditional hope expressions will evaluate organizational future outlook as less pessimistic compared to those who do not.

## 3 Materials and methods

### 3.1 Data and sample

Data were collected from a leading Korean corporate review platform where current and former employees anonymously share organizational experiences, describing strengths, weaknesses, and suggestions for management in free-text format. In addition to overall satisfaction rated on a 5-point Likert scale, respondents also indicate their expectations for whether the company will grow, remain similar, or decline in the following year.

The total collected dataset comprised 466,645 reviews. To implement the “fixed current satisfaction” design central to this study, a sub-sample of 93,276 reviews with a current overall satisfaction rating of exactly 2 out of 5 was selected for analysis. This restriction allows estimation of the pure effects of textually reflected psychological mechanisms on future outlook, by examining variance in future outlook within a group that is uniformly dissatisfied yet variable in their expectations for the future.

Methodologically, this study employs what we term a “controlled observational” design. By restricting the analytic sample to employees who share the same level of current satisfaction (rating of 2 out of 5), we effectively hold constant a key confound that pervades most satisfaction and outcome research. This approach functions analogously to a quasi-experimental design in which current satisfaction serves as a naturally occurring “treatment” that is held fixed, allowing us to examine variance in the outcome (future outlook) attributable to other factors, specifically, linguistic markers of helplessness and hope. While this design does not fully eliminate endogeneity concerns (e.g., unobserved individual differences may jointly influence language use and future outlook), it substantially reduces confounding relative to designs that allow current satisfaction to vary freely.

The selection of a satisfaction rating of 2 out of 5 as the focal subsample warrants justification. A rating of 2 represents a meaningfully dissatisfied group (below the scale midpoint) while avoiding the floor effect associated with the lowest rating of 1, where extreme dissatisfaction may reflect qualitatively different experiences (e.g., immediate exit intentions or hostile work environments). The rating of 2 captures employees who are clearly dissatisfied yet still engaged enough to articulate their experiences in review texts, making them an ideal population for examining the cognitive mechanisms that differentiate future outlooks among the dissatisfied. Additionally, this rating category contains the largest concentration of dissatisfied employees in the dataset, providing sufficient statistical power for detecting meaningful effects.

Prior to analysis, several text preprocessing steps were applied to ensure data quality. First, duplicate reviews and entries with fewer than 10 characters were removed. Second, Korean text was tokenized using morphological analysis to segment words into meaningful units. Specifically, a Korean morphological analyzer was employed to decompose compound words and identify base forms (lemmatization), which is essential for accurate dictionary matching given the agglutinative nature of the Korean language. Stopwords (common particles, conjunctions, and function words with minimal semantic content) were removed. However, conditional endings (e.g., ~다면, ~라면) were deliberately retained as they constitute key linguistic markers in this study. For the BERT embedding analysis, the raw text was used without lemmatization or stopword removal, as transformer-based models are designed to process natural language in its original form and can leverage contextual information from all tokens.

### 3.2 Variable measurement

#### 3.2.1 Dependent variable: organizational future outlook

The dependent variable (next_year_rating) captures respondents' assessments of what they expect for their organization in the following year, classified into three ordered categories: 1 = growth, 2 = similar, and 3 = decline. In the analytic sample, the distribution was approximately 7% growth, 53% similar, and 40% decline.

#### 3.2.2 Independent variable 1: helplessness score

The helplessness_score measures linguistic patterns related to learned helplessness in the text. Measurement employed a hybrid approach combining dictionary-based analysis and semantic embedding analysis.

First, a helplessness vocabulary dictionary was constructed, grounded in Abramson et al.'s (1978) reformulated learned helplessness theory and Peterson et al.'s (1993) attributional style research. According to these frameworks, the core of learned helplessness lies in attributing negative outcomes to stable, global, and uncontrollable causes. Korean language expressions reflecting these three attributional dimensions were identified: (1) stability attribution, including terms meaning “originally,” “unchanged,” “always,” “forever,” indicating that problems are perceived as enduring rather than temporary (Peterson and Seligman, 1984); (2) uncontrollability attribution, including expressions meaning “anyway,” “useless,” “meaningless,” “can't change,” reflecting the absence of contingency between action and outcome (Maier and Seligman, 2016); (3) globality attribution, including expressions such as “just,” “hopeless,” “gone,” indicating that problems are pervasive rather than domain-specific; (4) resignation and giving up, including vocabulary such as “give up,” “no expectations,” “no hope,” reflecting the motivational consequences of helplessness (Abramson et al., 1978).

Second, using the pre-trained multilingual sentence embedding model (paraphrase-multilingual-MiniLM-L12-v2), semantic similarity analysis was conducted. Seed sentences representing helplessness (e.g., “nothing will change anyway,” “there's no point in saying anything,” “this organization has always been like this”) were defined, and cosine similarity between each review text and the seed sentences was calculated (helpless_embed). The final helplessness score was computed by standardizing both the dictionary-based score and the embedding-based score to z-scores and averaging them.

To clarify the scoring procedure explicitly: the dictionary-based component was scored using relative word frequency. Specifically, for each review text, the dictionary score was calculated as the proportion of dictionary terms appearing in the text relative to the total number of tokens in that review. No additional word weighting scheme (e.g., TF-IDF or subjective importance weights) was applied; all dictionary terms were treated with equal weight. This approach follows the standard practice in dictionary-based text analysis, where the frequency of theoretically relevant terms serves as the primary indicator of construct presence (Tausczik and Pennebaker, 2010). For the BERT embedding component, each review text was encoded into a dense vector representation using the pre-trained multilingual sentence embedding model, and cosine similarity was computed between the review vector and the average vector of predefined seed sentences. The resulting cosine similarity value served as the embedding-based score. The two component scores (dictionary-based proportion and embedding-based cosine similarity) were each standardized to z-scores and then averaged with equal weight to produce the final composite score. This hybrid approach was adopted because dictionary-based scoring captures explicit lexical markers of the target construct, while the embedding-based scoring captures broader semantic similarity that extends beyond exact word matches, thereby compensating for the inherent vocabulary limitations of any finite dictionary.

#### 3.2.3 Independent variable 2: hope score

The hope_score was measured using the same hybrid approach as the helplessness score. The hope vocabulary dictionary was constructed based on Snyder's (2002) hope theory and Luthans et al.'s (2007) PsyCap research. Vocabulary reflecting the two core components of hope theory, namely pathway thinking and agency thinking, was selected: (1) pathway thinking vocabulary, including terms such as “direction,” “method,” “institution,” “structure,” “process,” indicating awareness of means for achieving goals (Snyder et al., 1991); (2) agency thinking vocabulary, including terms such as “will,” “attempt,” “effort,” “drive,” indicating beliefs about motivation and ability to execute pathways; (3) change possibility vocabulary, including terms such as “change,” “transform,” “improve,” “innovate,” “reform,” indicating belief that the current state can change (Carver and Scheier, 2002); (4) enhancement and improvement vocabulary, including terms such as “strengthen,” “supplement,” “reorganize,” “introduce,” indicating awareness of specific improvement actions.

The construction and validation of the helplessness and hope dictionaries followed a systematic process. Initial word lists were derived deductively from the theoretical frameworks described above (Abramson et al., 1978; Snyder, 2002). The author, a bilingual researcher with expertise in organizational psychology, systematically reviewed the candidate terms for relevance and face validity through multiple iterative rounds. The final dictionaries were then tested against a random subsample of 500 reviews to assess coverage and precision. To assess the psychometric properties of the dictionaries, we computed internal consistency and split-half reliability. The helplessness dictionary (15 terms across 4 subcategories) yielded a Cronbach's α of 0.080 (subcategory-level) and a mean Spearman-Brown corrected split-half reliability of 0.092 (SD = 0.016). The hope dictionary (23 terms across 4 subcategories) yielded a Cronbach's α of 0.212 (subcategory-level) and a mean split-half reliability of 0.203 (SD = 0.020). These values are lower than the conventional thresholds for survey-based scales (Nunnally, 1978). However, it is important to contextualize these metrics. Traditional internal consistency measures assume parallel indicators of a single latent construct, whereas text analysis dictionary terms represent diverse linguistic expressions spanning different theoretical facets (e.g., stability, uncontrollability, globality, and resignation for helplessness). Moreover, the inherent sparsity of text data (where most individual terms appear in fewer than 5% of reviews) structurally limits inter-item correlations (Grimmer and Stewart, 2013). Low internal consistency is a well-documented characteristic of dictionary-based text analysis tools, including widely used instruments such as LIWC (Tausczik and Pennebaker, 2010). Critically, this limitation is precisely why the present study adopted a hybrid measurement approach: the BERT embedding component captures semantic meaning beyond exact word matches, compensating for the inherent limitations of dictionary-based scoring alone. The criterion validity analysis confirmed that the helplessness dictionary score was significantly positively correlated with pessimistic future outlook (r = 0.020, p < 0.001), consistent with the theoretical direction, while the combined dictionary-embedding score used in the main analyses demonstrated substantially stronger predictive power.

An additional consideration regarding the low reliability levels concerns the potential influence of text preprocessing on dictionary matching accuracy. Although morphological analysis and lemmatization were applied to the text data for dictionary-based scoring (as described in Section 3.1), the reliability remained low even after this preprocessing step, which we acknowledge as a limitation of the current measurement approach. The agglutinative nature of the Korean language means that perfect lemmatization is difficult to achieve, and some morphological variants may not have been fully reduced to their base forms. This discrepancy between the “formal” and “structured” terms in the dictionary and the more colloquial, varied expressions in the actual review data may have contributed to lower inter-item correlations and, consequently, reliability estimates that fall well below the conventional threshold of α ≥ 0.70 (Nunnally, 1978). This low level of internal reliability is also reflected in the criterion validity coefficient: the relatively low Pearson'sr = 0.020 for the criterion validity of the helplessness dictionary score, despite its statistical significance (p < 0.001), is consistent with the low internal reliability and warrants careful interpretation. The statistical significance of this correlation is likely attributable in large part to the massive sample size (N = 93,276), which provides sufficient statistical power to detect even very small effect sizes as statistically significant (Cohen, 1992). While the significant p-value reflects consistency with the theoretical prediction (i.e., the direction of association is as expected), the small magnitude of the correlation (well below the conventional reliability benchmark of r ≥ 0.70) indicates that the dictionary-based score alone captures only a limited portion of the variance in future outlook. This reinforces the rationale for the hybrid measurement approach, where the BERT embedding component substantially augments the dictionary's predictive capacity. Readers should note that the reliability and validity estimates reported here are specific to the present study's context—Korean organizational reviews on an online platform—and may not directly generalize to other cultural, linguistic, or organizational settings. Future research applying these dictionaries in different contexts should re-evaluate their psychometric properties and be attentive to the cultural and organizational boundaries that shape how employees express helplessness and hope linguistically.

#### 3.2.4 Independent variable 3: conditional expression flag

Whether the text contains conditional expressions was measured as a binary variable (conditional_flag). Conditional expressions are the grammatical manifestation of pathway thinking in hope theory. According to (Snyder 2002), the core of pathway thinking is conditional reasoning of the form “if A, then B is possible.” In Korean, such reasoning is clearly marked by specific verbal endings corresponding to “if/when,” “if [hypothetical],” and “if [counterfactual].” If the relevant pattern appeared at least once in the text, it was coded as 1; otherwise as 0. Approximately 30% of the analytic sample included conditional expressions. As (Tausczik and Pennebaker 2010) noted, the use of conditional language is associated with causal reasoning ability and future-oriented thinking, reflecting analytical understanding of the situation beyond mere emotional expression.

#### 3.2.5 Control variable: message length

Since longer texts have a higher probability of containing specific language patterns, the natural logarithm of message length (log_message_length) was included as a control variable.

### 3.3 Analytical strategy

Because the dependent variable, organizational future outlook, is an ordinal variable measured in three ordered categories, namely growth (1), similar (2), and decline (3), ordered logit regression was used as the primary analytical method (Long and Freese, 2014). In the ordered logit model, a positive coefficient indicates that as the variable increases, the probability of being classified in a higher category (i.e., more pessimistic future outlook) increases. A negative coefficient indicates increased probability of being classified in a lower (more optimistic) category.

To complement the ordered logit coefficients, which are expressed in log-odds units and difficult to interpret intuitively, average marginal effects (AME) were calculated to assess the substantive impact of each independent variable on the probabilities of the three outcome categories (Williams, 2012). For continuous variables, the effect of a one standard deviation increase was calculated; for binary variables, the effect of a change from 0 to 1 was calculated.

Several additional robustness analyses were conducted: (1) binary logit models at different cutpoints to test the proportional odds assumption (Brant, 1990); (2) a multinomial logit model relaxing the ordinal assumption; (3) Winsorizing (trimming the top and bottom 1%) of continuous independent variables to control for extreme values; and (4) an interaction model with current employment status (current vs. former employee) to examine potential heterogeneity.

## 4 Results

### 4.1 Descriptive statistics

Among the 93,276 organizational members with a current satisfaction rating of 2 out of 5, the distribution of future outlook was: growth (next_year_rating = 1), approximately 7%; similar (2), approximately 53%; and decline (3), approximately 40%. This distribution empirically confirms the core premise of the study: even among employees with equivalently low current satisfaction, future outlooks diverge markedly. Approximately 7% believe “things are hard now but the organization will grow next year,” while approximately 40% expect decline.

Regarding key independent variables, hope scores and helplessness scores were standardized with mean = 0 and standard deviation ≈ 0.7. The mean of the conditional expression flag was 0.30, indicating that approximately 30% of reviews included conditional expressions such as “if [something happens].”

### 4.2 Hypothesis testing: ordered logit results

The results of the core ordered logit analysis are presented in Table 1.

**Table 1:** Ordered logit regression results predicting organizational future outlook.

Variable	*b*	*SE*	*z*	*p*
*Hope_score*	−0.004	0.010	−0.44	0.660
*Helplessness_score*	0.110	0.009	12.04	< 0.001
*Conditional_flag*	−0.072	0.015	−4.95	< 0.001
*Log_message_length*	0.149	0.014	10.44	< 0.001

N = 93,276. Log-Likelihood = −83,036. Dependent variable: future outlook (1 = growth, 2 = similar, 3 = decline). Positive coefficients indicate higher probability of more pessimistic outlook. b = unstandardized ordered logit coefficient. SE, standard error.

Regarding Hypothesis 1, the coefficient for helplessness_score was b = 0.110, which was statistically significant (p < 0.001). This positive coefficient indicates that employees who use more resignation language (e.g., “nothing will change anyway,” “this organization has always been like this”) have a higher probability of expecting organizational decline. This result strongly supports Hypothesis 1.

Regarding Hypothesis 2, the coefficient for conditional_flag was b = −0.072, also statistically significant (p < 0.001). This negative coefficient indicates that the use of conditional expressions is associated with an increased probability of being classified in a lower (more optimistic) outcome category. Employees who use conditional expressions such as “if things change, it will get better” tended to hold less pessimistic views of their organization's future compared to those who did not. This supports Hypothesis 2.

An intriguing and unexpected finding was that hope_score was not statistically significant (p = 0.660). The simple use of positive, hopeful vocabulary such as “improvement,” “change,” or “innovation” was not directly associated with future outlook. Yet, as confirmed above, conditional expressions, the linguistic manifestation of pathway thinking, showed a significant effect. This contrasting pattern provides empirical support for Snyder's (2002) contention that hope is not a simple positive emotional state but a cognitive capacity for imagining specific pathways from the current state to a desired goal.

### 4.3 Effect sizes: average marginal effects

Since ordered logit coefficients are expressed in log-odds units, average marginal effects (AME) were calculated to assess the substantive influence of each variable. Results are presented in Table 2.

**Table 2:** Average marginal effects on probability of each outcome category.

Variable	Δ*P* (Growth)	Δ*P* (Similar)	Δ*P* (Decline)
*Helplessness_score (+1 SD)*	−0.52%p	−1.46%p	+1.98%p
*Hope_score (+1 SD)*	+0.02%p	+0.05%p	−0.07%p
*Conditional_flag (0 → 1)*	+0.49%p	+1.23%p	−1.72%p

AME, average marginal effect expressed in percentage points (%p). For continuous variables (+1 SD), the effect of a one standard deviation increase is shown. For conditional_flag (0 → 1), the effect of moving from non-use to use is shown.

A one standard deviation increase in helplessness score increases the probability of a decline outlook by approximately 2 percentage points. Conversely, using conditional expressions decreases the probability of decline outlook by approximately 1.7 percentage points. Although these effect sizes may appear modest at the individual level, when applied across a workforce of 465,000 employees, they carry considerable organizational-level significance. For instance, widespread use of conditional hope expressions could shift tens of thousands of employees from “decline” to “similar,” or from “similar” to “growth” in their outlook.

### 4.4 Scenario analysis

To provide more intuitive understanding of the results, predicted probabilities were calculated for combinations of helplessness levels (−1 SD, mean, +1 SD) and conditional expression use (0, 1), as presented in Table 3.

**Table 3:** Predicted probabilities by helplessness level and conditional expression use.

Conditional expression	Helplessness	*P* (Growth)	*P* (Similar)	*P* (Decline)
No	Low (−1 SD)	7.4%	53.9%	38.7%
No	Mean	6.9%	52.5%	40.7%
No	High (+1 SD)	6.3%	51.0%	42.7%
Yes	Low (−1 SD)	7.9%	55.1%	37.0%
Yes	Mean	7.3%	53.7%	39.0%
Yes	High (+1 SD)	6.8%	52.3%	40.9%

Predicted probabilities were derived from the ordered logit model, holding all other variables at their means.

Table 3 clearly illustrates the interplay between helplessness and conditional hope expression. The lower the level of helplessness and the more conditional expressions are used, the lower the probability of a decline outlook. For example, the probability of decline outlook is 42.7% for those with high helplessness (+1 SD) who do not use conditional expressions, compared to 37.0% for those with low helplessness (−1 SD) who do use conditional expressions, a difference of approximately 5.7 percentage points.

### 4.5 Asymmetric effects of hope score

To better understand why hope_score was non-significant in the ordered logit model, additional binary logit analyses were conducted. Results revealed an asymmetric pattern: (1) binary logit for growth (1) vs. rest (2/3): b = 0.103, p < 0.001 (significant); (2) binary logit for decline (3) vs. rest (1/2): b = 0.013, p = 0.205 (not significant).

These results indicate that hope language affects the choice of a “growth” outlook but not the distinction between “decline” and “similar.” In other words, hope language is associated with active optimism (“the organization will grow”) but not with passive avoidance of pessimism (“it will not decline”). In contrast, helplessness_score showed significant effects in both binary logit models, indicating a consistent negative influence across the full spectrum of future outlooks.

### 4.6 Robustness checks

Several robustness analyses confirmed the stability of the main findings. First, multinomial logit analysis relaxing the proportional odds assumption yielded consistent results: helplessness_score significantly increased the probability of choosing the “decline” category (b = 0.216, p < 0.001), and conditional_flag significantly decreased this probability (b = −0.066, p = 0.031).

Second, after Winsorizing continuous independent variables (top and bottom 1%), both helplessness_score (b = 0.116, p < 0.001) and conditional_flag (b = −0.071, p < 0.001) effects remained significant with comparable magnitude, confirming that results are not driven by extreme values.

Third, analysis of the interaction between helplessness_score and current employment status (current vs. former employee) revealed that the main effect of current employment was significant (b = 0.195, p < 0.001), indicating that current employees tend to hold more pessimistic outlooks than former employees. However, the interaction effect was not significant (b = −0.004, p = 0.848), suggesting that the negative effect of helplessness language on future outlook operates similarly across both groups.

## 5 Discussion

### 5.1 Interpretation of main findings

The findings of this study can be summarized along three main lines. First, the use of helplessness language is significantly associated with pessimistic organizational future outlook. Second, the use of conditional hope expressions mitigates pessimism in future outlook. Third, simple hope vocabulary use alone shows no significant relationship with future outlook.

The first finding supports the application of learned helplessness theory (Seligman, 1975) to the organizational context. Expressions such as “nothing will change anyway” and “this organization has always been like this” indicate that organizational problems are being attributed to stable and uncontrollable causes, and such attribution patterns lead to pessimistic future expectations. This is consistent with the dynamics of organizational helplessness proposed by (Martinko and Gardner 1982) and empirically demonstrates that helplessness language exerts substantial influence on future perceptions beyond mere emotional expression.

The second finding connects to Snyder's (2002) hope theory, particularly the concept of “pathway thinking.” The conditional expression “if things change, it will get better” indicates awareness of a specific pathway from the current situation to improvement. Such pathway thinking is distinct from vague optimism and connects to a cognitive evaluation of the possibility of change. The observation that users of conditional expressions hold less pessimistic future outlooks reflects their perception of the organizational environment as controllable.

The third finding, that simple hope vocabulary use is unrelated to future outlook, is counterintuitive but important. Positive vocabulary such as “improvement,” “change,” and “innovation” does not necessarily signal an optimistic future outlook, because such vocabulary can also be used in contexts pointing to current problems (e.g., “improvement is needed”). This underscores the importance of considering the contextual meaning of words in text analysis and echoes warnings by (Grimmer and Stewart 2013) about the pitfalls of context-free bag-of-words approaches.

### 5.2 Theoretical implications

This study makes several theoretical contributions to organizational behavior research.

Temporal extension of psychological contract theory. Whereas, prior research has focused on contract violation/fulfillment at the current time point, this study highlights the temporal dimension of the psychological contract by exploring how future outlooks form. Whether organizational members believe that “the contract will be maintained in the future” may exert important influence on their current behaviors and attitudes, an aspect underexplored in existing literature.

Integrative application of hope theory and learned helplessness theory. This study competitively tested these two theoretical frameworks in the context of organizational future outlook and simultaneously documented the direct negative effect of helplessness and the buffering effect of conditional hope expressions. This suggests that employees' future perceptions are formed through two complementary mechanisms: inhibition of negative attribution (helplessness) and activation of positive pathway thinking (conditional hope). This integration advances both theories by demonstrating their differential and concurrent relevance in an organizational field setting.

Language as a window into cognition. This study examined at scale, across approximately 465,000 naturally occurring reviews, how linguistic patterns are associated with cognitive states (future outlooks). In particular, the discovery that conditional expressions can function as a linguistic marker of pathway thinking extends the possibilities of language-based organizational perception research and aligns with the broader tradition of computerized text analysis in organizational and psychological research (Tausczik and Pennebaker, 2010; Berger et al., 2020).

The findings also carry implications for understanding the “waiting culture” of Korean organizations. Korean organizational contexts have traditionally been characterized by norms of deferred gratification, an implicit promise that endurance will eventually be rewarded (Hofstede, 2001; House et al., 2004). This cultural pattern may moderate short-term dissatisfaction and sustain organizational stability. However, this study reveals the dual character of such a waiting culture. When waiting leads to actual improvement, it represents the realization of “deferred hope.” But when waiting is repeatedly frustrated, it converts into “learned helplessness.” Once the perception that “waiting won't make a difference anyway” spreads, the entire organization may fall into a state of collective resignation. Furthermore, the waiting culture can become a tool of exploitation: when the message “bear with it; it will get better” is repeated without actual improvement, it perpetuates current unreasonableness by exploiting employees' deferred hope. The strong association between helplessness language and pessimistic future outlook found in this study warns that such exploitation will ultimately result in “contract exhaustion” and abrupt organizational departure.

These findings also resonate with prior research conducted in Korean organizational settings. Studies on Korean employee attitudes have consistently demonstrated that cultural factors such as collectivism, hierarchical deference, and long-term orientation significantly shape how employees evaluate their organizational experiences (Jung and Yoon, 2015; Lee and Kim, 2023). Recent research has further shown that career growth opportunities play a critical role in determining turnover intentions through organizational commitment (Lee et al., 2019), underscoring the importance of future-oriented perceptions in the Korean employment context. The strong association between helplessness language and pessimistic future outlook observed in this study may be particularly pronounced in Korean organizations, where hierarchical decision-making structures can amplify perceptions of uncontrollability. When employees perceive that organizational outcomes are determined solely by top management with little input from below, the stable and uncontrollable attributions characteristic of learned helplessness may become self-reinforcing. Conversely, the buffering effect of conditional hope expressions suggests that Korean employees who can identify specific, actionable pathways for change, despite operating within hierarchical constraints, maintain more resilient future outlooks. This aligns with research on proactive behavior in East Asian organizational contexts (Shin and Kim, 2015), which suggests that even in high power-distance cultures, pathway thinking can function as a cognitive resource for sustaining engagement.

### 5.3 Practical implications

Making change pathways visible. This study found that conditional expressions mitigate pessimism. Management should present improvement plans clearly and specifically, framing commitments in conditional form (“when X is completed by Y, Z will improve”) rather than offering vague assurances (“we will do better”). This approach explicitly maps the pathway from current condition to future improvement, which our findings suggest is the linguistically and cognitively effective form of hope communication.

Preventing the spread of helplessness. Once the resignation of “nothing will change anyway” spreads within the organization, it becomes a self-fulfilling prophecy: helplessness leads to abandonment of attempts; abandonment of attempts leads to actual absence of change; this reinforces helplessness. Management should proactively share cases in which employee opinions have actually been reflected and make even small changes visible, thereby restoring “action and outcome contingency” in employees' minds.

Managing deferred hope. Deferred hope is a double-edged sword. It helps people endure current difficulties, but when repeatedly frustrated, it converts into contract exhaustion. Before telling employees to “hang on; things will get better,” organizations must honestly assess whether they are prepared to fulfill that promise. An unfulfilled promise is the surest way to transform hope into helplessness.

### 5.4 Limitations and future research directions

Cross-sectional design. Because this study analyzed review data collected at a single time point, it is difficult to determine whether the use of helplessness language “causes” pessimistic future outlooks or whether pessimistic employees are more likely to “use” helplessness language. As (Podsakoff et al. 2003) noted, the possibility of common method bias cannot be ruled out in cross-sectional designs, although the use of different measurement approaches for the independent variables (text-based scores) and the dependent variable (future outlook rating) partially mitigates this concern. Rigorous causal inference requires longitudinal or experimental designs; (Ployhart and Vandenberg 2010) emphasized that causal inference in organizational research requires longitudinal data with at least three time points.

Sample representativeness. Employees who leave reviews on an online corporate platform may not represent all Korean workers. (Landers and Behrend 2015) identified self-selection bias as a limitation of online data collection, and individuals with extreme experiences may be more likely to write reviews (Hu et al., 2009), potentially leading to distributional polarization. Nevertheless, the large-scale sample of over 465,000 reviews may partially offset these biases, and recent research confirms that online review data demonstrates considerable convergent validity with traditional survey data (Stamolampros et al., 2019).

Text measurement limitations. Although this study employed a hybrid methodology combining dictionary-based analysis and BERT embeddings, it remains difficult to fully capture meaning changes depending on context. For example, “change is necessary” and “change is possible” include the same vocabulary but carry different meanings. As (Grimmer and Stewart 2013) noted, all text analysis methods have limitations in fully capturing the complexity and context-dependence of language. Moreover, since the dictionaries were constructed by researchers based on theory, expressions of helplessness or hope not included in the dictionaries may have been missed.

Relatedly, the measurement approach may be subject to construct contamination, as some dictionary terms may carry ambiguous contextual meanings (e.g., “change is needed” vs. “change is possible” both contain the word “change” but reflect different orientations). Future studies should consider manual annotation of a representative subsample to validate the automated dictionary-based classifications against human judgment. Such validation would strengthen confidence in the correspondence between linguistic markers and the theoretical constructs they are intended to capture. Additionally, the emerging field of text-mining-based organizational research (Piwowar-Sulej et al., 2023; Hannigan et al., 2019; Yu and Xiang, 2023) offers sophisticated methodological frameworks (including topic modeling, structural topic models combined with word embeddings, and advanced natural language processing pipelines) that could complement and extend the hybrid approach employed here.

In particular, the rapid advancement of large language models (LLMs) presents promising opportunities for future research. Beyond the BERT embeddings used in this study, more recent LLMs (e.g., GPT-4, LLaMA) could enable more nuanced sentiment analysis, zero-shot classification of hope and helplessness expressions, and context-sensitive interpretation of conditional language (Brown et al., 2020). LLM-based approaches could also facilitate cross-lingual validation of the linguistic constructs identified here, enabling researchers to test whether the associations between helplessness language, conditional hope expressions, and future outlook generalize beyond the Korean language context.

Context-specificity of dictionary-based text analysis. More broadly, text-based analysis using predefined dictionaries can be a powerful tool when applied within a well-defined context, but its effectiveness diminishes when applied across contexts with blurred or heterogeneous boundaries. The dictionaries developed in this study were constructed based on theoretical frameworks and validated within the specific context of Korean organizational reviews on an online platform. The linguistic expressions of helplessness and hope captured by these dictionaries are shaped by the cultural norms, organizational practices, and communication conventions particular to this setting. Consequently, applying the same set of dictionaries to different cultural contexts (e.g., Western organizational settings), different industries, or different types of text data (e.g., internal surveys, exit interviews) may yield substantially different results. As (Pennebaker et al. 2015) noted in the development of LIWC, dictionary-based tools reflect the linguistic conventions of the contexts in which they were developed and validated. Future research and practitioners should therefore exercise caution when transferring these dictionaries to other settings and should be aware of the cultural and organizational context boundaries that constrain the generalizability of the present findings. Ideally, researchers should recalibrate or reconstruct dictionaries when applying text-based analysis to new cultural or organizational contexts.

Absence of organizational-level controls. Employees' future outlooks may be influenced by organizational-level factors such as actual performance, industry environment, organizational size, and leadership. As (Kozlowski and Klein 2000) emphasized, organizational phenomena are inherently multilevel, and individual-level analysis alone cannot capture the full picture. While this study partially controlled for organizational-level effects by holding current satisfaction constant, explicit multilevel analysis was not conducted and remains an important direction for future work.

Simplicity of the future outlook measure. The dependent variable (next_year_rating) was measured in three categories (growth/similar/decline). While useful for capturing employees' future outlooks, it does not distinguish the multidimensionality of future orientation, for example, overall organizational performance vs. individual career outlook, or short-term vs. long-term outlook (Parker et al., 2010).

Future research directions that address these limitations include: (1) longitudinal studies to examine causal mechanisms, particularly event-study designs tracking how employees' language patterns and future outlooks change before and after specific organizational events (restructuring, leadership changes, performance announcements); (2) testing the predictive validity of future outlook for behavioral outcomes such as turnover (Griffeth et al., 2000), organizational citizenship behavior, and job performance; (3) experimental studies on organizational interventions, such as comparing the differential effects of conditional-form communication (“when X is done, Y will improve”) vs. generic promises (“we will improve”) on employee future outlook, building on the PsyCap intervention approach of (Luthans et al. 2010); and (4) advanced text analysis utilizing large language models (LLMs) to more precisely capture the subtle linguistic nuances of helplessness and hope (Brown et al., 2020).

## 6 Conclusion

This study analyzed responses from approximately 465,000 Korean employees to the question: “Things are hard now; will next year be different?” The core finding is as follows: even among employees with equivalently dissatisfied present circumstances, future outlooks diverge dramatically. At this bifurcation point, helplessness language (“nothing will change anyway”) amplifies pessimism, and conditional hope expressions (“if X changes, things will improve”) mitigate it. What matters is not the simple use of positive vocabulary, but the capacity to imagine pathways to change. This is the essence of hope as theorized by (Snyder 2002).

These findings carry an important message for organizations. More dangerous than current difficulties is the extinction of future expectations. The moment employees believe “nothing will change anyway,” the psychological contract has already been exhausted. Organizations must ensure that their members can believe “change is possible,” and must ensure that such beliefs are not frustrated. If you ask people to wait, you must prove that the waiting is worthwhile.

## Data Availability

Publicly available datasets were analyzed in this study. The data were collected from a publicly accessible corporate review platform. All data were anonymized and contained no identifiable personal information.
